# Supplementary figures and images for: Campylobacter jejuni motility integrates specialized cell shape, flagellar filament, and motor, to coordinate action of its opposed flagella
Source: PLoS Pathog. 2020 Jul 2;16(7):e1008620. doi: 10.1371/journal.ppat.1008620 (PMC7332011; doi:10.1371/journal.ppat.1008620)

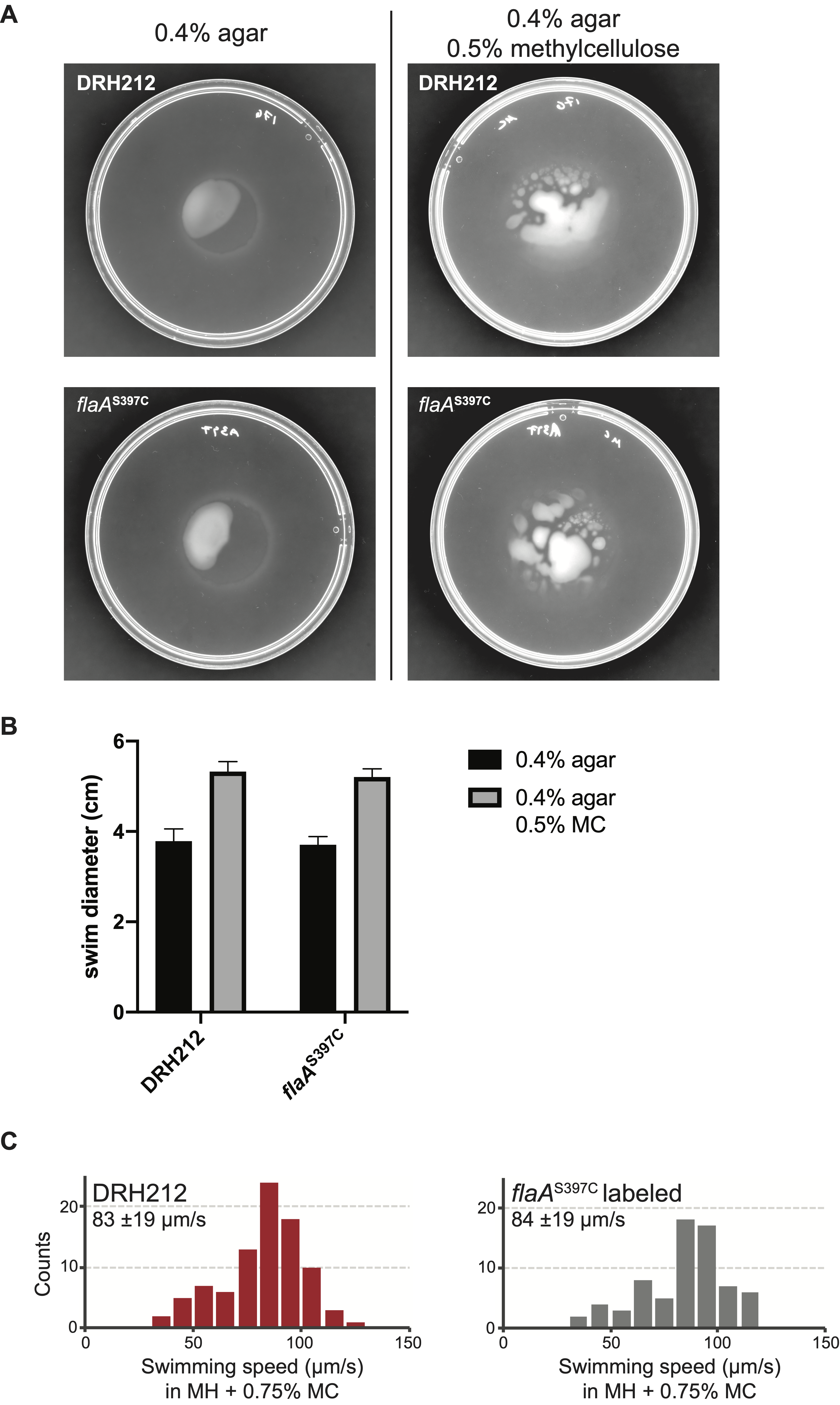

Supplement: S1 Fig — Motility plates with or without methylcellulose added were inoculated with either DRH212 or EJC28 and allowed to incubate for ~24 hours. In both low-viscosity and high-viscosity motility agar, EJC28 were found to swim as well as DRH212 (A and B). Swim halo diameters in B are the average of five replicates, with error bars representing the SEM. To determine whether swimming velocity was impacted in EJC28 relative to DRH212, and whether labeling with DyLight and FM 4–64 had an effect on swimming, cultures of DRH212 and labeled cells were observed with 20x objective lens phase contrast microscopy. Both DRH212 and labeled EJC28 were found to swim at comparable velocities (C), demonstrating that neither the flaAS397C mutation itself nor fluorescent labeling impacts swimming ability. (TIFF) [file ppat.1008620.s001.tiff]

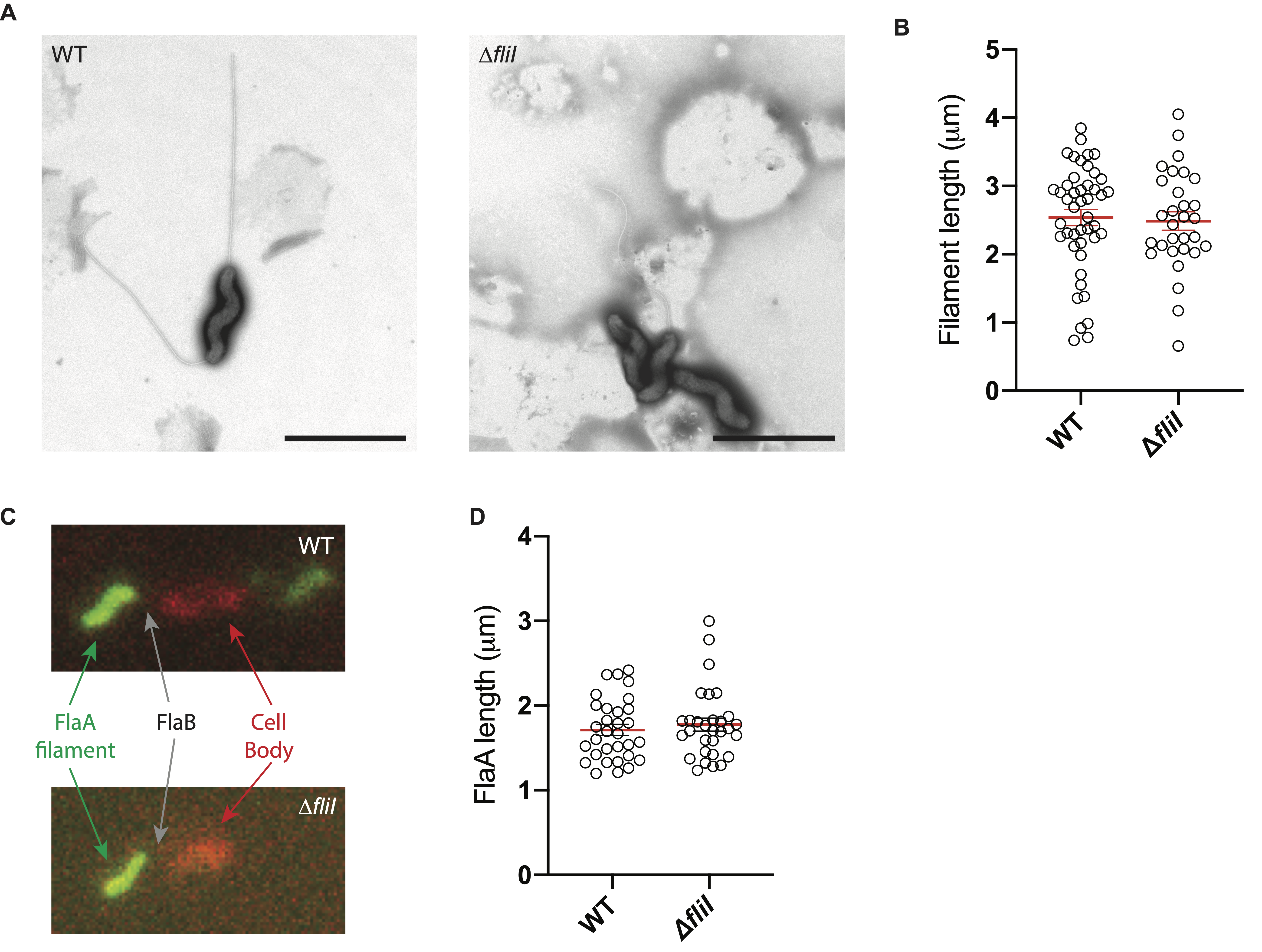

Supplement: S2 Fig — Cells of EJC28 and ΔfliI (EJC30) were applied to formvar-coated TEM grids, negative stained with phosphotungstic acid and observed at 4,400x magnification (A). While the ΔfliI mutant constructed fewer flagella/cell, flagellar length was comparable to WT (B). The flagellar filaments of EJC30 were observed to have an unlabeled FlaB portion similar in length to the WT (C), and the fluorescent FlaA filament for both WT and EJC30 were found to be approximately the same (D), indicating that the composition of the flagellar filament of the ΔfliI mutant is similar to WT. For B and D, filament length was determined by measuring by hand in ImageJ (FIJI) and plotted in Graphpad Prism, with error bars representing the SEM. For B, 45 WT filaments and 30 ΔfliI filaments were measured. For D, 31 WT filaments and 30 ΔfliI filaments were measured. (TIFF) [file ppat.1008620.s002.tiff]

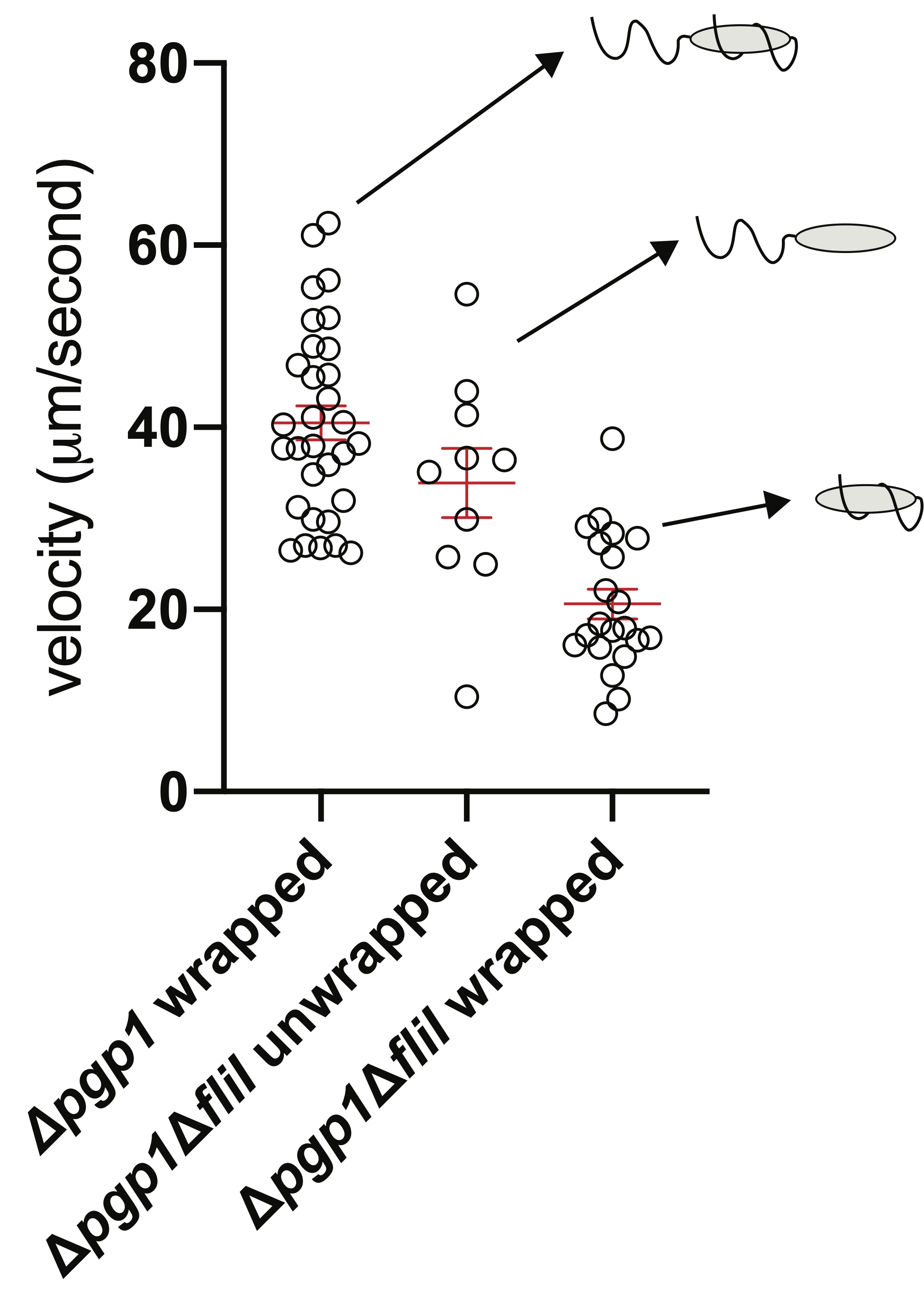

Supplement: S3 Fig — fliI was deleted in the Δpgp1 straight-cell background in order to determine how much a helical cell body shape contributes to propulsion in high viscosity media. Similar to the ΔfliI pgp1+ helical strain (Fig 1F and S4 Movie), singly-flagellated straight cells were found to be slower than doubly-flagellated straight cells, with the singly-flagellated wrapped cells being the slowest of the three. (TIFF) [file ppat.1008620.s003.tiff]

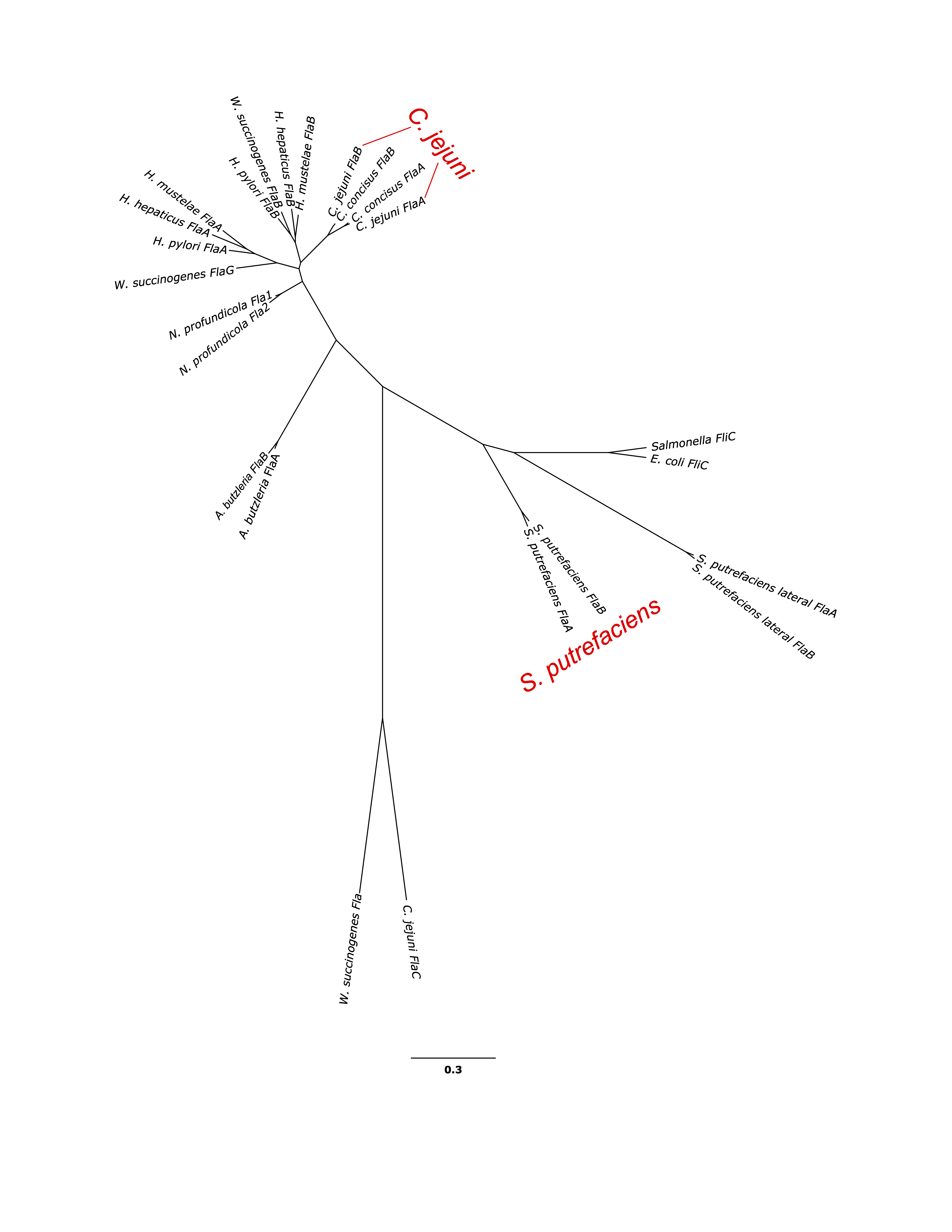

Supplement: S4 Fig — Sequences of the conserved N- and C-terminal flagellin regions were aligned using FSA; unconserved gaps were removed using T-coffee; and the phylogeny was determined using RAxML and visualized with FigTree. (TIFF) [file ppat.1008620.s004.tiff]

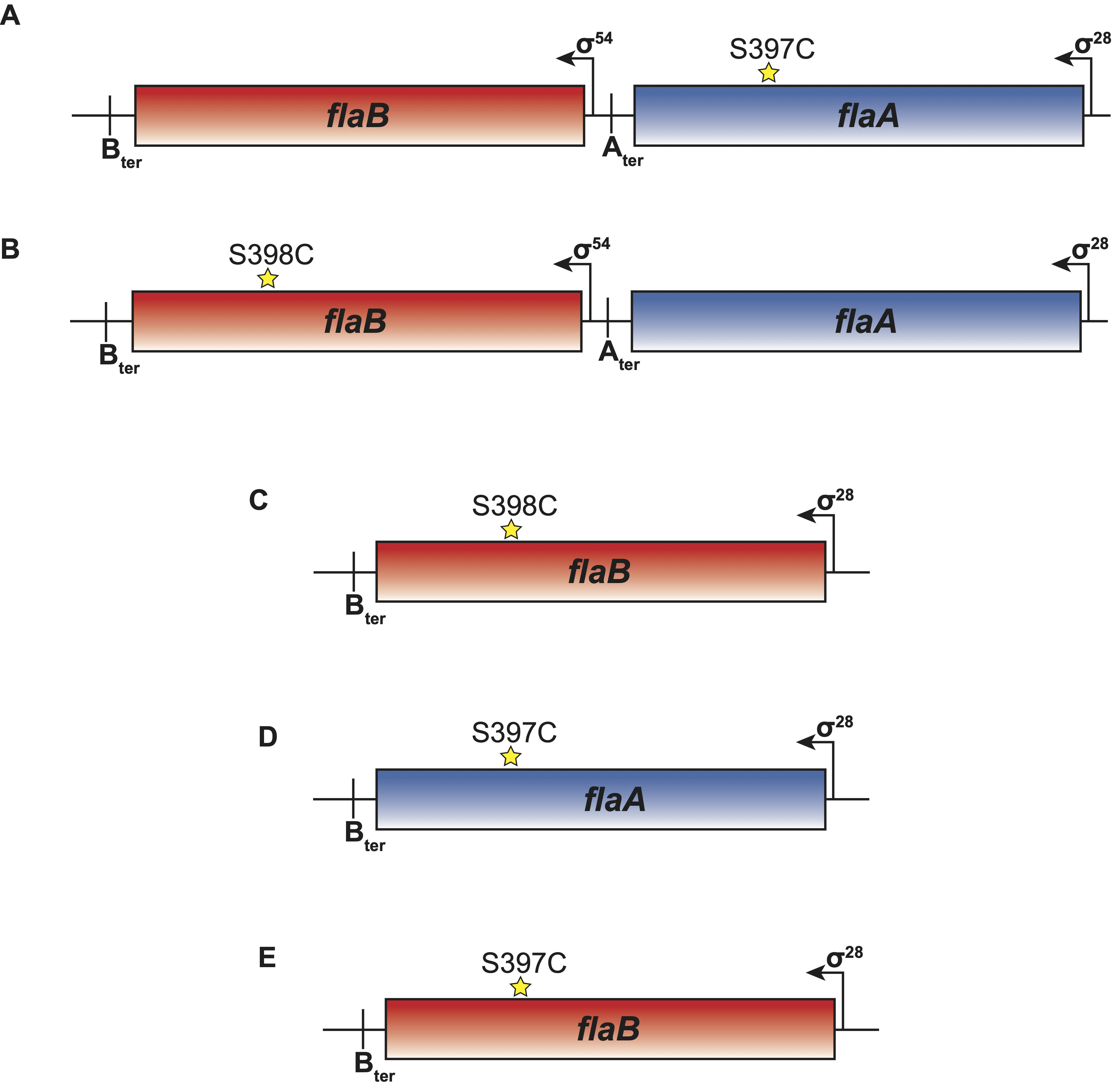

Supplement: S5 Fig — With the exception of strain WPK440 (S3 Movie), all fla cysteine alleles generated for this study were chromosomally encoded at the native flaAB locus. The WT strain for this study, EJC28 (flaAS397C), has a WT copy of flaB expressed from its native σ54 promoter (A). Our original flaB cysteine allele, flaBS398C, (B and C, S16 and S17 Movies) was found to label poorly relative to the S397C allele. Consequently, for flow chamber experiments (Fig 5, S18 and S19 Movies), we generated a single-flagellin mutants, harboring either flaAS397C or flaBS397C at the fla locus. In each case, the flagellin is expressed from the flaA σ28 promoter (D and E). (TIFF) [file ppat.1008620.s005.tiff]

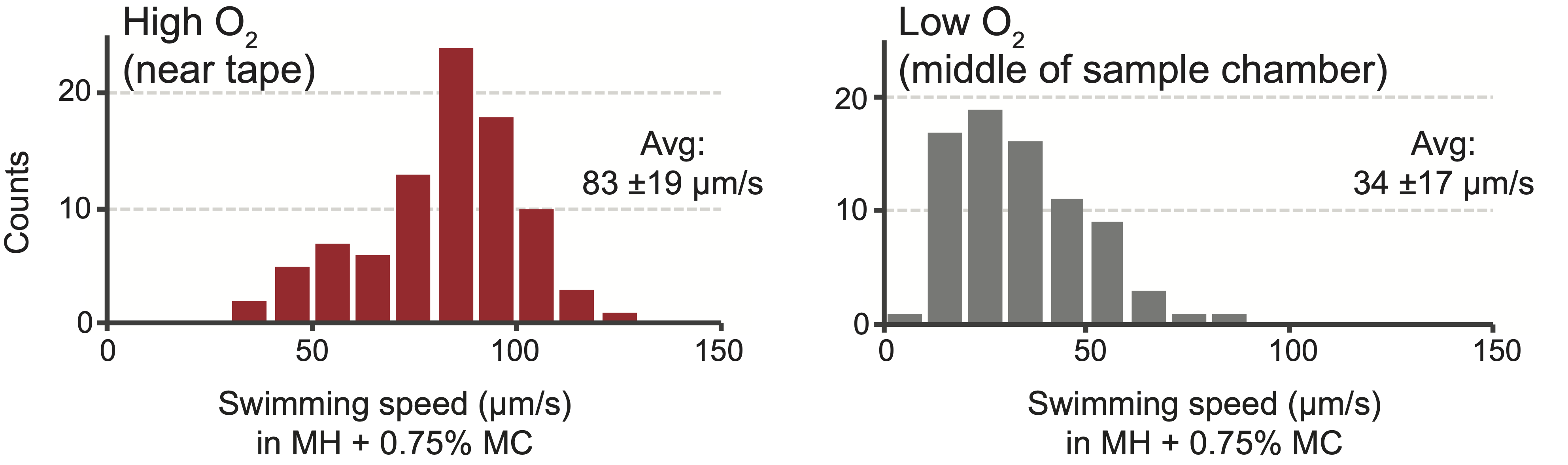

Supplement: S6 Fig — When cells were tracked using 20x magnification phase-contrast microscopy (no fluorescent labeling), cells that were in the middle of the sample chamber swam at approximately half the velocity of cells near the taped edges of the sample chamber. This is presumed to be due to lower oxygen concentration in the middle of the sample chamber compared to near the porous, double-sided tape used to construct sample chambers, leading to a reduced proton motive force (PMF) to drive flagellar motor rotation. (TIFF) [file ppat.1008620.s006.tiff]

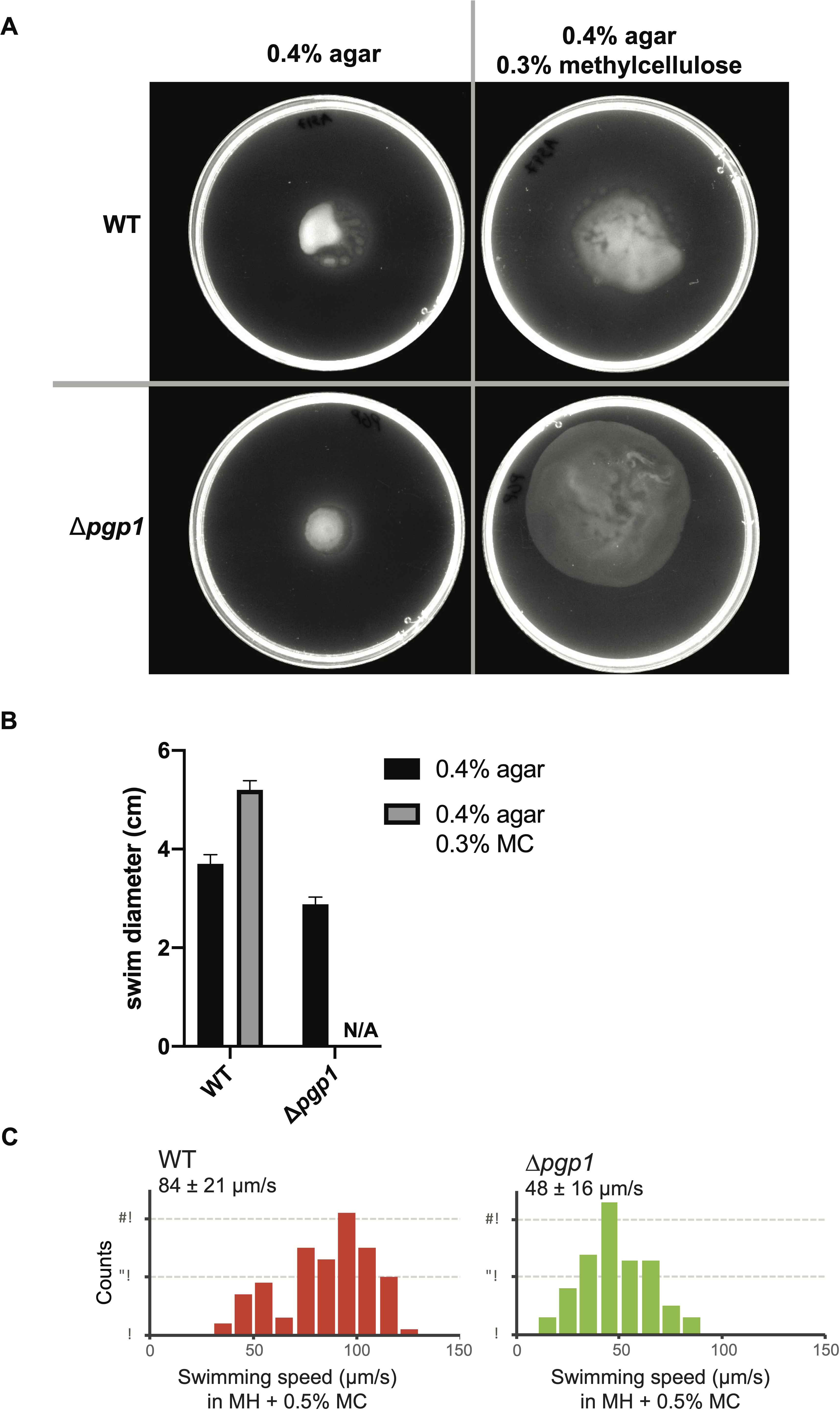

Supplement: S7 Fig — In regular motility agar (MH + 0.4% agar) the Δpgp1 mutant was found to swim nearly as well as WT, as judged by the diameter of the swim halo (2.88 cm vs. 3.70 cm, respectively. Values are mean of 5 replicates for each with error bars representing the SEM). In high-viscosity motility agar (MH + 0.4% agar + 0.3% methylcellulose (MC)), however, the Δpgp1 mutant was found to be incapable of penetrating and swimming through the agar. Rather, the straight cell mutant spread across the surface of the media (A and B). Using low magnification (20x) phase contrast microscopy, Δpgp1 cells in MH + 0.5% MC were found to swim at ~50% the velocity of WT cells, as has been previously reported. (TIFF) [file ppat.1008620.s007.tiff]

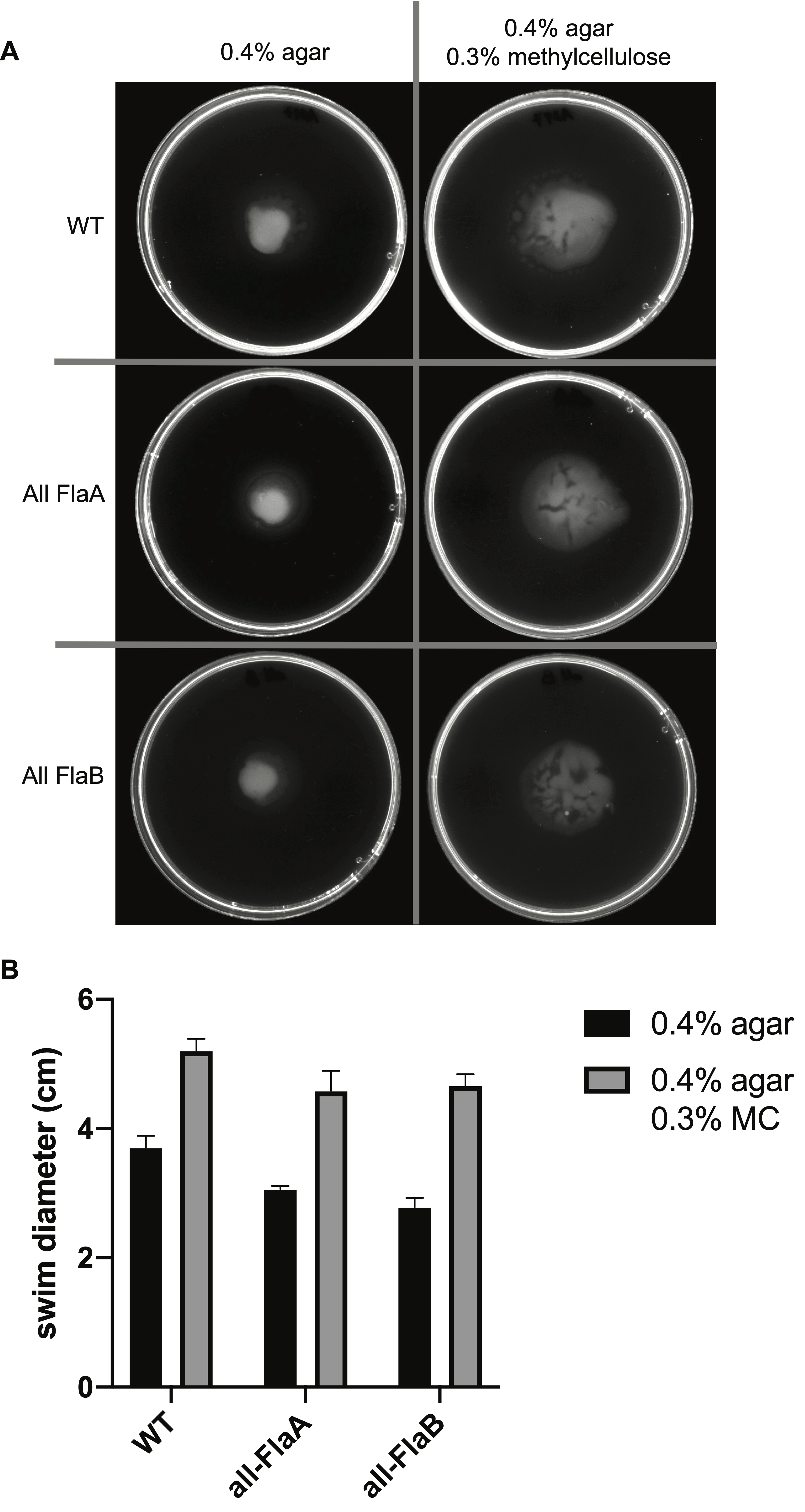

Supplement: S8 Fig — In both regular and high-viscosity motility agar, the all-FlaA and all-FlaB mutants were found to swim with comparable efficiency, but both are inferior to WT with its composite filament assembled from both flagellin types (A and B). Values in B are the average of 5 replicates for each strain and condition, with error bars representing the SEM. (TIFF) [file ppat.1008620.s008.tiff]
