# Supplementary material for: Campylobacter jejuni motility integrates specialized cell shape, flagellar filament, and motor, to coordinate action of its opposed flagella
Source: PLoS Pathog. 2020 Jul 2;16(7):e1008620. doi: 10.1371/journal.ppat.1008620 (PMC7332011; doi:10.1371/journal.ppat.1008620)
Supplement: Attachment — Submitted filename: Response to reviewers.docx [file ppat.1008620.s030.docx]

**Reviewer #1:**

**Although I have completely understood usage of "wrapped" and "unwrapped" in the text, I felt uneasy with usage in some sentences.
For example, 163: filament is wrapping the cell body but not wrapped.160: wrapped filament.**

*The text has been changed to more clearly communicate which filament is wrapped and which is unwrapped for these examples*

**2. 172. It is better to use the pitch distance and the diameter of a helix than pitch angle, which is often used in engineering and particle physics.**

*While we agree with the reviewer that pitch distance and helix diameter are usually better suited for a biological audience, in this case we would prefer to retain our usage of pitch angle, because we wish to emphasize that the angle of the cell body and flagellar filaments are approximately opposite. We believe that this point is an important evolutionary facet of our story as a mechanism to reduce contact between the flagellar filament and the cell body. We can change it if the reviewer and/or editor insist, but would otherwise prefer to leave it as-is.*

**Reviewer #2:**

**A key question is whether the leading flagellum provides propulsive force. I do not understand the authors' line of reasoning for why they conclude that it does. Cells with wrapped flagella move faster, but couldn't this be an inverse causation: what if high speed causes wrapping?**

*We reached this conclusion based on two observations: 1) monoflagellated cells with a wrapped (leading) filament are motile, demonstrating conclusively that the wrapped flagellum is propulsive, and 2) presence of wrapped (leading) filaments speed up cells: dual-flagellated cells with a wrapped filament and unwrapped (lagging) filament swim faster than (up to twice the speed) monoflagellated cells with only an unwrapped (lagging) filament. Therefore the wrapped filament contributes up to one half of the propulsive force during wrapped mode swimming. Furthermore the cells we observed swimming at maximum velocity (80-100 um/sec.) are those with two flagella, one wrapped and one unwrapped. This is inconsistent with the leading (wrapped) flagella providing no propulsive force: we would expect monoflagellated, unwrapped cells to swim at or near maximum velocity.*

*We can rule out high speed itself causing wrapping, as we have observed wrapped cells in conditions that cause low speed swimming. For example, cells in high concentrations of Ficoll or cells observed in the middle of the sample chamber swim much slower than those under our normal conditions, but wrapping still occurs for both. Furthermore, in the rare cases of paralyzed flagella, these flagella never wrap, suggesting wrapping is (at least partially) driven by an active flagellum.*

*Wrapping of the leading filament does not occur only once a threshold velocity has been reached. During directional switches (i.e. chemotaxis), the cell halts momentarily, but wrapping occurs within several milliseconds of a directional change, before the cell is translating at high speed. This is also true for monoflagellated cells transitioning from unwrapped- to wrapped-mode swimming during chemotaxis. This shows that wrapping is dependent on motor torque (and the viscosity of the environment), but that velocity itself is relatively unimportant for wrapping.*

**2. The authors use several mutants to try to address questions like flagellar number of cell shape, but they assume that these mutants do not disrupt other properties of the cell. For example, the straight mutant affects cell wall - what if it affects cell stiffness, which would also affect speed? Or if the flagella of fliI mutants are weaker?**

*The reviewer is correct to point this out, and we have added disclaimers to the manuscript to reflect the possibility that our Δpgp1 and ΔfliI mutants may have characteristics that we haven’t accounted for that may affect our results (see lines 136-138 and 253-256 of the revised manuscript).*

*For the* Δ*pgp1 mutant, we are unable to determine whether the biophysical properties of the cell body (e.g. cell stiffness) have been impacted. This is an important question, but at this time we are unable to return to the lab to carry out experiments. Nevertheless, we have never observed qualitative changes of shape in any of our already-acquired videos. We expect that experiments to study the effects of the* Δ*pgp1mutation on, e.g., cell-body stiffness may be technically challenging, beyond our combined expertise, or require development of new methodologies. This, among other questions, will be a focus of future planned work examining the* C. jejuni *cell body and peptidoglycan sacculus more broadly.*

*Similarly, although we cannot rule out that the* Δ*fliI filaments are weaker than WT filaments, that* Δ*fliI filaments are as long as WT filaments strongly suggests that the* Δ*fliI mutation does not weaken the filament. We would expect a weaker filament to be more likely to break during swimming, which would lead to* Δ*fliI cells having shorter filaments on average. Additionally, we have not found any precedent in the literature to suggest that defects in the FliHIJ ATPase or T3SS as a whole cause defects in the biomechanical properties of the extracellular hook/filament, although it is possible. See, for example, the work of Marc Erhardt in Salmonella* fliI *mutants.*

**Reviewer #3: None.**

**Part III – Minor Issues: Editorial and Data Presentation Modifications**
Please use this section for editorial suggestions as well as relatively minor modifications of existing data that would enhance clarity.


**Reviewer #1: 126: what does "stored" mean?
386: E.coli and Salmonella should be in Italic.
423: "yank" may be "pull".
448: typo, facilate.**

*Thank you for the suggestions, we have made the requested changes to the revised manuscript.*

**Reviewer #2: I really enjoyed this paper and think it would be a valuable contribution to understanding the biophysical basis of bacterial motility. However, I wonder if its best home is really PLoS Pathogens. If the editor and authors think yes, then I am highly supportive. I just hope it will reach the right audience, as opposed to something like Biophysical Journal.**

*Thank you, we are happy to publish in PLoS Pathogens as long as the editor(s) feels it is appropriate.*

**Reviewer #3:**

**Abstract, line 19: Enterobacteriaceael? Perhaps rephrase to ‘the norm of Enterobacteriaceae’?**

*We have made this change to the manuscript, thank you for the suggestion.*

**Single flagellated cells. The authors used a DfliI mutant, which assembles flagella less frequently and thus most cells have none or only one flagellum. While Fig. S2 shows that assembled flagella have approximately the same length, can the authors exclude that the ratio of the two flagellins (FlaA/FlaB) is not changed, which would result in different properties of the filament? This might also account for the observed decreased motility?**

*The reviewer is right to point this out. Although we cannot completely rule out that the distribution/ratio of FlaA and FlaB along the filament is changed in the ΔfliI mutant relative to WT, re-analysis of our data strongly supports our assumption that deletion of* fliI *does not affect the ratio. We have measured the lengths of the FlaA filaments in our* Δ*fliI movies as well as the FlaA filaments in movies of WT cells. There was no significant difference in lengths between the two (~1.7 um for each). This was done by manually tracing the fluorescent signals in ImageJ and comparing the lengths of ~30 FlaA filaments for each. Additionally, the non-fluorescent FlaB “gaps” between the dyed cell bodies and fluorescent FlaA filaments are discernible for both WT and* Δ*fliI, and appear to be approximately the same, although we did not attempt to measure FlaB length. We have added our measurements of fluorescent FlaA filaments to figure S2 as well as images of WT and* Δ*fliI highlighting the cell body, FlaA filaments and the location of FlaB as a gap in fluorescence.*

*There are obvious limitations to this approach. For one, it is possible that there is a mixture of some non-labeled FlaB in the labeled FlaA filament, which we cannot detect. Conversely, there could be a small amount (i.e. below the limit of detection) labeled FlaA in the FlaB stub, although the fact that there is a non-labeled gap between the filament and body (i.e. FlaB) suggests that the composition of the filament is at least similar to WT. Finally, since we are measuring low-resolution 2D images of cells in 3D space, the filament lengths reported are shorter than those taken by TEM, where the filaments lay flat on the grid and can be measured much more accurately. These issues could be addressed biochemically or by making new mutants, but we are currently unable to get into the lab and do not know when we will be able to. Nevertheless, we are confident that the overall ratio remains approximately the same after* fliI *deletion.*

**Are there any cells in the WT, which only produce one flagellum? Even if there are only a few, it might be useful to compare motility of these uniflagellated cells with uniflagellated cells of the DfliI mutant.**

*We thank the reviewer for this suggestion. It’s a great idea. We have gone through our videos of WT cells and were able to find several monoflagellated cells, both unwrapped and wrapped and calculated their swimming speed and compared to* Δ*fliI cells.*

*For the unwrapped (lagging) monoflagellated WT cells, the velocity is similar (~50 um/second) the* Δ*fliI* mutant*. For the wrapped (leading) monoflagellated WT cells, the velocity is actually higher than the corresponding* Δ*fliI cells. But the trend (i.e. unwrapped is faster than wrapped) still holds for monoflagellated cells, with monoflagellated WT cells being significantly slower than dual-flagellated WT cells.*

*We realized, however, that there is a major caveat to measuring the swimming velocities of “mono-flagellated” WT cells: we can’t exclude the possibility of there being a non-labeled filament, or portion of a filament, on these cells.*

*In a typical experiment, the cell suspensions were resuspended in MH broth following labeling and placed back in the incubator and aliquots were taken as needed for observation for up to 30-45 minutes afterwards. This would be more than enough time for a cell that had only one filament prior to labeling to construct a partial or full filament. So, the results from our WT “monoflagellated” cells must be taken with a grain of salt.*

**3) The DcheY mutant experiment to demonstrate that the default rotational direction of the motor is CCW is a beautiful example of how simple genetics can answer a fundamental problem.**

*Thank you! We thought long and hard about the best possible controls, so it’s nice to see our investment appreciated…*

**4) Does flagellin glycosylation contribute to the observed cell body wrapping of the filament? Is flagellin in the flaA_S397C mutant still glycosylated?**

*We did not determine whether the filaments of the flaA_S397C mutant were still glycosylated, but we assume that they are due to previous work done by* Guerry et al. Guerry et al. *have shown that mutants that are entirely unable to glycosylate FlaA are unable to construct filaments, are non-motile and are also defective for autoagglutination (AAG). Additionally, when specific serine codons in* flaA *are mutated (and are no longer able to be glycosylated), filament assembly and motility are severely reduced.*

*There are 19 serines in FlaA that have been shown to be glycosylated, including S397. Of these 19 serines, eight have an impact on either motility or AAG, while the remaining positions show no discernible impact on either motility or AAG. S397 was chosen for our study because it was likely to be surface exposed and is among the eleven serine residues that show no impact on motility or AAG when mutated to an alanine.*

*Since our S397C mutant constructs full length flagella and displays WT motility, we feel we can rule out any defect in glycosylation having an impact on our observations.*

**5) Concerning the question of how unwrapped cells are able to swim in low-viscosity media (i.e. both flagella at the opposing cell poles are unwrapped and would cancel each other’s propulsion). One possibility is that – contrary to the proposed model – the motors at the opposing cell poles rotate in different directions. Did the authors analyze the swimming behavior of their DcheY mutant in low-viscosity media? This would at least ensure that both flagella are rotating CCW and might support alternative hypotheses, e.g. that different filament lengths or helical pitches enable swimming of unwrapped cells.**

*We have videos of the* Δ*cheY mutant swimming in low-viscosity media and they have the same phenotype as WT, with the exception that we see no reversals. We still see ~50% being wrapped in low-viscosity media. As with WT, we see unwrapped* Δ*cheY cells that are still able to swim. We can include video of the* Δ*cheY mutant in low-viscosity media as a supplemental video if requested.*

**6) Line 384 + 396: define the abbreviations ‘LH’ and ‘RH’ in the manuscript text.
7) Line 386: E. coli and Salmonella should be italics
8) Fig. 1B y-axis should be labeled (e.g. ‘proportion of cells’)
9) Fig. 2: The rotational direction of flagella and the cell body is indicated by arrows. Consider to use different colors for the arrows indicating rotational direction of flagella and the cell body. Also, for the first schematic, both the top flagellum and cell body are indicated to rotate CCW. This is wrong for the cell body, which should rotate CW?
10) Fig. 3: Bad quality of the schematic and figure labels
11) Fig. 4A: Scale bar is missing.
12) Fig. 4B, D, E: Should read ‘∆pgp1’
13) Fig. 5: Bad quality of the schematic and figure labels
14) Fig. 1B, 4E, 5C, S1B, S2B, S7B, S8B: Information concerning the number of replicates or samples, as well as error bars are missing and should be indicated in the figure legend.
15) Fig. S2A+B: Should read ‘∆fliI’.
16) Fig. S2B + S2: y-axis label should read ‘µm’
17) Fig. S7: Bad quality of the figure labels**

*Thank you for pointing these out, we have made the requested changes to the manuscript and added the requested information to the figure legends concerning number of replicates, sample number etc. We have also changed the color of arrows and labeling of figure two to make it more clear to the reader.*
